# P56. A novel cancer vaccine with nanogel-based antigen transporter and sequence-optimised long peptide antigen

**DOI:** 10.1186/2051-1426-2-S2-P30

**Published:** 2014-03-12

**Authors:** N Harada, D Muraoka, T Hayashi, F Momose, H Shiku, Y Tahara, S Sawada, K Akiyoshi

**Affiliations:** 1 Mie University Graduate School of Medicine, Department of Cancer Vaccine, Tsu Mie, Japan; 2 Kyoto University Graduate School of Engineering, Department of Polymer Chemistry, Kyoto, Japan

## Background

The limited success of cancer vaccines in clinical could be owing to their low efficacy that can be largely affected by their formulation. Vaccine formulation usually consists of three components, antigen, delivery system, and adjuvant. Recently, long peptide antigen (LPA) has been attracting interest, because (1) one LPA can provide antigen presenting cells (APCs) with multiple T cell epitopes and (2) it avoids undesired presentation by non-professional APCs that can lead to the exhaustion of antigen-specific T cells. While a LPA can include multiple epitopes, the prerequisite design of junction of epitopes (inter-epitope sequence, IES) for robust immunogenicity is not yet established, and we explored it in this study. On the other hand, accumulating data has been suggesting the importance of delivery system for cancer vaccines. By employing a novel nanogel-based delivery system, we tried to improve the immunogenicity of LPA vaccine.

## Materials and methods

LPA was designed to include three mouse cytotoxic T cell (CTL) epitopes that were linked with the IES of oligotyrosine, oligothreonine, oligoglycine or oligoproline. The complex of LPA and cholesteryl pullulan (CHP) nanogel was fabricated, and subcutaneously injected to mice with TLR agonist such as CpG oligoDNA or poly-IC RNA as an adjuvant. Frequency of vaccine-induced CTLs specific to each epitope was then determined by intracellular IFN-γ staining assay. Using similar mouse model, the effect of CHP nanogel delivery system on transportation of LPA to APCs in the draining lymph node (DLN) as well as T cell response to vaccination was also examined, comparing with a conventional delivery system, incomplete freund adjuvant (IFA).

## Results

The IES candidates were selected based on in silico prediction of the sensitivity to proteasomal cleavage. The sensitivity of oligotyrosine and oligothreonine was predicted high, while that of oligoglycine and oligoproline was low. In the vaccinated mice, the LPA including the oligotyrosine IES most robustly stimulated specific CTLs towards all three epitopes included in LPA. The LPA including the oligothreonine IES was second best. In contrast, LPA including the oligoglycine or oligoproline IES often induced very limited CTL response. In the evaluation of delivery system, the CHP nanogel transported LPA to APCs in the DLN much more efficiently than IFA did. The kinetics of the CHP nanogel-mediated LPA transportation was closely similar to that of APC activation by TLR agonists, suggesting that the CHP nanogel harmonises the action of LPA and adjuvant. Indeed, in the presence of TLR agonists, the CHP nanogel greatly augmented the immunogenicity and anti-tumour effect of LPA vaccine.

## Conclusions

We succeeded in the enhancement of immunogenicity of LPA vaccine by the rational design of IES in LPA and the CHP nanogel-mediated efficient antigen transportation to the DLN. These technologies will bring a remarkable improvement of vaccine efficacy.

